# The relationship between mood disorders, personality disorder and suicidality in adolescence: does general personality disturbance play a significant role in predicting suicidal behavior?

**DOI:** 10.1186/s40479-023-00238-9

**Published:** 2023-11-01

**Authors:** Riccardo Williams, Marco Chiesa, Marta Moselli, Camillla Frattini, MariaPia Casini, Peter Fonagy

**Affiliations:** 1 Department of Dynamic and Clinical Psychology and Health Studies, Faculty of Medicine and Psychology, “Sapienza” - University of Rome, Rome, Italy; 2 Research Department of Clinical, Educational and Health Psychology, University College London, London, UK; 3 Section of Child and Adolescent Neuro-Psychiatry, “Sapienza” – University of Rome, Rome, Italy

**Keywords:** Personality disorders, Personality dimensions, Adolescence, Suicide, Mood disorder

## Abstract

**Introduction:**

Current research points to the importance personality pathology and Major Depression e as relevant psycopathological risk factors for understanding suicidal risk in adolescence. Literature has mainly focused on the role of BPD, however current orientations in personality pathological functioning suggest that BPD may be the representative of a general personality disturbance, a factor of vulnerability underlying diverse psychopathological variants and aspects of maladaptive functioning. However, recent studies seem to have neglected the contributions that other specific personality disorders and personality pathology as a general factor of vulnerability for suicidality; and only marginally investigated the interaction of personality disorder (PD) as an overall diagnosis and individual PDs and major depression (MDD).

In this paper, the independent and cumulative effects of MDD and DSM-IV PDs on suicidal risk are investigated in a sample of adolescents observed in a longitudinal window of observation ranging from three months preceding the assessment to a six-month follow up period of clinical monitoring.

**Methods:**

A sample of 118 adolescents (mean age = 15.48 ± 1.14) referred for assessment and treatment on account of suicidal ideation or behavior were administered the CSSRS, SCID II, Kiddie-SADS at admission at inpatient and outpatient Units. All subjects included in the study had reported suicidal ideation or suicide attempts at the C-SSRS; The CSSRS was applied again to all patients who reported further suicidal episodes during the six-months follow-up period of clinical monitoring. Dimensional diagnoses of PDs was obtained by summing the number of criteria met by each subject at SCID-%-PD 5, In order, to test the significance of the associations between the variables chosen as predictors (categorical and dimensional PDs and MD diagnosis), and the suicidal outcomes variables suicide attempts, number of suicide attempts and potential lethality of suicide attempt, non-parametric bivariate correlations, logistic regression models and mixed-effects Poisson regression were performed PD.

**Results:**

The categorical and dimensional diagnosis of PD showed to be a significant risk factors for suicide attempt and their recurrence, independently of BPD, that anyway was confirmed to be a specific significant risk factor for suicidal behaviors. Furthermore, PD assessed at a categorical and dimensional level and Major Depression exert an influence on suicidal behaviors and their lethality both as independent and cumulative risk factors.

**Limitations:**

Besides incorporating dimensional thinking into our approach to assessing psychopathology, our study still relied on traditionally defined assessment of PD. Future studies should include AMPD-defined personality pathology in adolescence to truly represent dimensional thinking.

**Conclusion:**

These results point to the importance of early identification of the level of severity of personality pathology at large and its co-occurrence with Major Depression for the management of suicidal risk in adolescence.

## Background

### The impact of suicidality in adolescence

Suicide is a remarkable public health problem worldwide and its reduction has been declared a primary objective by the World Health Organization [1]. It is the fourth leading cause of death among people between 14 and 29 years old worldwide [2–4]. In the USA and other Western Country suicide has proved to be the second cause of death in adolescence [5, 6]. Suicidal ideation and behaviors typically make their appearance in adolescence. Entering middle adolescence drastically increases the likelihood for the emergence of suicidal behaviors [7], although recent studies indicate that the lifetime peak of suicidal behaviors, as well as other relevant clinical features of impulsivity and emotional dysregulation should not be limited to adolescence to include at least young adulthood [8]. Still, follow-up data indicate that suicide attempts occurring in adolescence are significant predictors of the occurrence of such behaviors in adulthood [9].

With reference to the consequences of the COVID-19 epidemic, first research data indicate that rates of suicidal ideation and suicidal behaviors between the ages of 11 and 19 are significantly higher for some months of 2020 compared to 2019 [10]. Epidemiological data also show that girls have a higher risk of engaging in suicidal behaviors, and boys tend to attempt suicide with potentially more lethal consequences [11]. Attempted suicide is a more statistically widespread phenomenon than actual suicide in adolescence, with a prevalence of 8–10%; the highest reported attempted suicide rate is between 15–24 years of age and only the 17% of the adolescents who report suicidal thoughts then actually attempt suicide [7, 12].

### Modelling the study of suicidality in adolescence: the suicidal process and the related predictive variables

Current empirical approaches to the understanding and management of suicidal risk outlined a sequential process beginning before the actual suicidal behavior takes place and even before the suicidal ideation becomes conscious [13]. Most contemporary models of suicide agree in identifying the experience of a un unbearable psychological pain as a starting point of the suicidal process. This condition of *psychache* is able to trigger a sense of entrapment and helplessness, and suicide is then perceived as the only way to escape from this unbearable emotional state [14]. This staging model of the suicidal process is supposed to begin with a motivational phase in which the early idea of one’s own death is gradually transformed into the more stable suicidal ideation, possibly leading to the proper suicidal intention and planning [15]. Many subjects report having nurtured suicidal ideation, and even intent, without having resorted to suicidal behavior, though. This is why many authors believe that the staging model of suicide should consider a volitional phase subsequent to the motivational one [15].

Provided this framework of understanding, clinicians and researchers are now working to identify specific risk factors or a configuration of risk factors that account for the passage from the appearance of early thoughts about one’s own death, to suicidal ideation and intention, then to real planning and execution of suicidal behavior. Research data show that only a relatively small percentage of subjects reporting suicidal ideation end up attempting suicide and even a smaller percentage actually succeed [16, 17]. In this regard, researchers have focused on the identification of those risk factors that differentiate individuals with suicidal ideation from those who attempt suicide [18]. A recent and acknowledged line of research has further refined the investigation of these volitional aspects of suicidality by specifically focusing on the relative impact of the risk factors on distinct suicidal variables [19]. In this study we adopted this research approach that specifically identifies the suicidal variables of suicidal ideation, occurrence of any suicide attempt, recurrence or number of suicide attempts, their potential lethality [20].

### Psychopathological risk factors for suicidality in adolescence: the role of affective disorders and personality disorders

Current research has evidenced that many risk factors may contribute to the emergence of suicidal ideation and conducts in adolescence. Specific risk factors concerning adolescence include both the developmental conditions of the brain [21] and an array of psychosocial stressors including, among others, traumatic experiences, family conflicts, victimization [22–24].

Research both in adulthood and adolescence has identified the substantial convergence of several psychopathological risk factors implicated in the transition from suicidal ideation to suicidal conducts. The emphasis has been especially put on mood disorders and personality pathological functioning (mainly associated with BPD) [18, 23].

### The impact of mood disorders on suicidality in adolescence

The significant association between affective disorder and suicide is well documented [25]. More specifically, the diagnosis of Major Depressive Disorder (MDD) and the occurrence of a depressive episode in Bipolar Disorder (BD) have been evidenced to play a major role for suicidal risk [26, 27]. The role of MDD and BD for suicidality has also been showed in adolescence [27]. The relevance of major depression and depressive symptoms as predictors of suicidality in adolescence is well established. Depression severity is in particular showed to be a strong predictor for suicide [28–30].

### The impact of personality disorders on suicidality in adolescence

The literature has also found a high prevalence of personality disorders in adolescent samples with suicidal ideation and behaviors [31].

A systematic review of studies of suicidal behaviors in adolescence has revealed that a diagnosis of personality disorder (PD) was associated with 19–23% of suicide attempts, and 30–42% of successful suicide [32]. Several contributions have suggested that a synergic interplay between external stressors and the presence of PDs, which may work as an activating factor, increases the risk for suicidal behavior [33–38]. PD may also play a facilitating role in turning suicidal ideation into suicidal behavior, by interfering with the effective processing of negative emotions and psychache, the state of mind supposed to trigger the suicidal process [39, 40]. In particular, several studies have found that the incidence of Borderline Personality Disorder (BPD) is high, ranging between 56 and 91%, in samples of suicidal adolescents and adults [41–43] and between 49 and 62% in attempted suicide [44, 45].

More recent investigations also aimed at identifying the contributions from other personality disorders besides BPD. In these studies, all Cluster B personality disorders were reported at higher risk for suicidal ideation and behavior, otherwise interpreted as variants of BPD [22]. More specific considerations concern the possible role of Narcissistic Personality Disorder (NPD) in the field of suicidality. Both in adulthood and adolescence NPD or narcissistic traits are reported to have apparently low or even inverse relationship with suicidal conducts [46].

However, clinical indications report that the peculiar aspects of the narcissistic pathological functioning are specific triggers for both suicidal ideation and unexpected but highly lethal suicidal conducts [46, 47].

Indeed, recent studies have confirmed that NPD plays a significant role in increasing the risk of recurrence of suicidal ideation and the lethality of suicidal attempts in adolescence [48] and adulthood [49–52].

Overall, current literature on personality disorders and suicidality in adolescence seems to have overlooked a more detailed investigation of the role of specific personality disorders other than BPD for suicidality [32]. Moreover, a quest for the understanding of the impact of personality pathology at large for suicidal conducts also deserves attention from the researchers. As evidenced later in this introduction, this quest seems justified in the light of the more recent debate on the diagnostic approach more suitable to detect personality disorders in adolescence and adulthood [53].

### The additive role of personality disorders and mood disorders on suicidality

Given the frequent co-occurrence of affective disorders and personality disorders [54], researchers have also investigated their mutually additive role in precipitating the suicidal conducts. There is a dearth of studies that tested the relative significance of PD and mood disorders separately and in combination in predicting the presence, severity and intensity of suicidal ideation and suicidal behavior. Some studies have indicated that specific BPD features can incrementally increase suicidality in subjects with depression, substance abuse and other psychopathologies [43, 55, 56]. For example, Sharp et al. [57] found that the presence of BPD compared to major depression (MDD) resulted in an increased suicidal ideation in a psychiatric sample of adolescents. Yalch et al. [58] considered the association between suicide risk and specific BPD features controlling for the effect of depressive symptoms, and found an independent incremental effect of identity disturbance and impulsivity on the observed variance in suicide risk scores [58]. Furthermore, empirical evidence highlights how aggression and impulsiveness are positively correlated with suicidal behavior only among BPD adolescents, whereas hopelessness and depression are positively correlated with suicidal behavior in both BPD and MDD diagnostic groups [59]. Other studies found that hopelessness and impulsive aggression independently increase the risk of suicidal behavior both in patients with BPD and with a major depressive episode [60]. A possible relationship between depression and narcissistic pathology has also been recently evidenced in adolescence [61].

While Soloff et al. [60] could not find any significant differences in the characteristics of suicide attempts between patients with BPD and those with MDD, patients with both disorders had significantly higher number of suicide attempts and degree of objective planning. In a group of borderline adolescents Mirkovic et al. [62] found a direct effect of MDD on lifetime suicidal attempts, and an indirect effect mediated by increasing emotional dysregulation, which may be an important risk factor for suicidal attempts in these patients. In another study, BPD patients with a history of MDD with melancholic features were more likely to have a history of suicide attempts compared to BPD with no MDD [63]. Subjects with comorbid BPD and MDD had a higher number of lifetime suicide attempts and made their first attempt at a younger age compared to subjects with BPD alone [64]. More specifically, it has been shown that BPD diagnosis mediates the impact of mood disorders on suicidal ideation and suicide attempts [56, 57]. The impact of each specific risk factor must be included within a model that conceives of suicide as the outcome of process in which the level of intentionality and the intensity of the ideation needs to be considered [13].

The present literature mainly focuses on combination of BPD with MDD in increasing the suicidal risk while little is known as to the association between MDD the presence of any PD or other specific PDs in predicting suicide in adolescence.

### New orientations in the understanding and diagnosis of personality pathology in adolescence

Notwithstanding the necessary caution in diagnosing personality disorders in adolescence, current orientations point to the importance of an early diagnosis, because of its relevance for the understanding of adolescents' maladaptive functioning, including suicidal behaviors and other aspects of disturbance this phase of life [65, 66]. The sources of criticism concerning the reliability, validity and clinical usefulness of such diagnoses have been gradually overcome in the face of emerging research evidence [65–70]. Although the validity of PD diagnoses in adolescence has been questioned, recent empirical studies indicate that the prevalence of PD diagnoses is similar between adolescence and adulthood [65]. Furthermore, the diagnoses of personality disorders in adolescence have overlapping clinical features with the personality disorders in adulthood [71, 72]. Finally, various studies have shown significant concurrent validity and predictive validity of PD diagnoses in adolescence with the clinical features of impulsivity, sexual promiscuous behaviors, substance abuse, aggressive conducts, disturbance of identity and social relationships [66, 73, 74].

Furthermore, the debate on the of diagnosis of PD in the adolescent population has been influenced by the wider issues concerning the approach to be employed in assessing personality disorders [71, 74]. In recent years there has been a long and significant [75, 76].

Debate on the way in which PD should be diagnosed, either as a categorical or a dimensional disorder. Although several studies have shown that a categorical approach may maintain its clinical validity in diagnosing the degree of severity of personality pathology [66, 77], a number of authors have argued that this model is undermined by excessive comorbidity, a degree of within-diagnosis heterogeneity, marked temporal instability, no clear boundary between normal and pathological personality pathology, and poor convergent and discriminant validity [72, 78, 79]. PD is therefore increasingly seen as a dimensional disorder, with emphasis on personality functioning and pathological personality traits [78, 80]. The continuity of personality pathology is attributed to core dimensions that are stable during development [81, 82]. The continuity of these core dimensions is due to both genetic and early environmental influences [81, 83]. These pathological personality traits can be accurately assessed in adolescence as outlined in the Alternative Model for Personality Disorders (AMPD) of the Section III of the DSM-5 [84, 85], and these traits seem to hold for the homotypic continuity of personality diagnoses in the lifespan [70]. Moreover, the dimensional diagnosis of PD of has been found to be a reliable predictor of maladjustment in adolescence and the stability of the diagnosis through to adulthood [65, 86].

The widely acknowledged need for early detection and intervention on personality disorders in adolescence [65] along with the shift in diagnostic orientations for personality pathology has thus led to prioritize the value of dimensional assessment of personality disorders in adolescence as well [71, 74]. At the same time, literature has also indicated the descriptive clinical advantages that may derive from the concurrent employment of the categorical and dimensional diagnoses of personality disorders [66, 77]. In particular, an integrated approach that includes categorical diagnoses of personality disorders seems to account for the degree of severity of the personality pathology and is predictive of the degree of social and behavioral maladjustment, increased affective dysregulation and, in particular, suicidal risk [87–89]. By virtue of these considerations, in this paper an integrated approach including both dimensional and categorical assessment of personality disorders has been adopted.

Many specific assessment approaches have been used to formulate a dimensional diagnosis of personality pathological functioning in adolescence [90–92].

In order to compound a dimensional and categorical assessment of personality disorders, we decided to employ the count of symptoms as derived from the SCID-5-PD for each personality disorder, a method that proved its clinical and statistical validity and reliability and temporal stability [72, 89, 93].

## Objectives of the study

In this study, we mean evaluate the importance of both categorical and dimensional PD diagnoses as well as MDD on suicidal behaviors and their potential lethality in adolescence. We report results of a prospective study of 118 adolescents followed up for 6 months after their first assessment at admission at day hospital and inpatient treatment Units. We, in particular, aim to evaluate the relative and cumulative significance of PD as a categorical and a dimensional construct meant to describe the degree of severity of personality pathology along with the diagnosis of MDD, in predicting suicidal behavior in terms of presence of any suicidal attempt, the number of suicidal attempts and the worst suicide episode potential lethality.

## Methods

### Study design and sample selection

One hundred twenty-eight adolescents aged between 12 and 18 years with either active suicidal ideation and/or a recent history of suicide attempt consecutively referred for admission to the day hospital and to the inpatient unit at a metropolitan Italian Pediatric Hospital between 2017–2019, were considered for study inclusion. Subjects with intellectual disabilities (IQ < 70) (*N* = 2), with severe impairment of adaptive and school functioning (*N* = 3) and a diagnosis of Autistic Spectrum Disorder according to the DSM-5 (*N* = 5) were excluded from the study. The remaining 118 subjects were assessed using a battery anamnestic and diagnostic self-report measures and semi-structured interviews. Subjects were confirmed as having active suicidal ideation if the Columbia Suicide Severity Rating Scale (C-SSRS) score was ≥ 2. 46 subjects out of the total of 118 had been admitted to the inpatient unit and 72 had been referred to the outpatient unit for the onset of a mood disorder or symptoms or behavioral problems.

A team of research psychologists and psychiatrists independent from the clinical teams were trained to reliability criteria on all measures through the use of original training videotapes. Each rater had regular supervision meeting with a senior psychiatrist, experienced in the delivery of the instruments used in the study. Coding and data entry were regularly monitored and adherence to protocol was checked using audiotapes and physical records. Each rater oversaw the administration and scoring of only one of the measures administered in the sample and was blind to the evaluations from the other measures.

All patients included in the study were regularly treated and monitored for six-months after admission. In particular, a clinical monitoring of the relapse of suicide attempts was carried out for all patients in a window of observation of six months after the admission. Notably, patients who reported suicidal ideation but no suicide attempt at admission showed no suicide attempt in the six months in the follow-up window of observation (we considered this sub-group of patients as *only*-*ideators*). Only two patients among the ones having attempted suicides at admission were reported to have another suicide attempt during the six-month follow-up window of observation (respectively one episode and two episodes, that when screened again with the CSSRS showed of lower level of potential lethality in comparison to the ones assessed at admission). The variable "number of suicide attempts" refers to the overall count of episodes throughout the whole window of observation (from three months prior the admission to six months of follow-up observation).

### Measures

General cognitive functioning was assessed through scaled tests based on age and language, including the *Raven Progressive Matrices Test* (Raven, 1981) and the *Wechsler Intelligence Scale for Children-Revised* (WISC-IV; Orsini, Pezzuti & Picone, 2012). The subjects’ intellectual abilities were classified according to the *Diagnostic and Statistical Manual of Mental Disorders*, 2000 (DSM-IV-TR).

*The Columbia Suicide Severity Rating Scale; Columbia University* (*C-SSRS;* [94]) is a scale that evaluate suicidal ideation in subjects aged twelve and over. The scale assesses the severity of suicidality in the domains of suicidal ideation and suicidal behavior. The C-SSRS rates four constructs: (a) The severity of the suicidal ideation, measured on a 5 points Likert scale (1 = desire to be dead; 2 = non-specific active suicidal thoughts; 3 = suicidal thoughts with a method; 4 = suicidal intent; 5 = suicide intent with a plan); (b) The intensity of the suicidal ideation is reckoned by investigating frequency, duration, degree of control, deterrents and reasons for the ideation; (c) Suicidal behavior rated for actual attempts, aborted attempts, preparatory acts and non-suicidal self-injury (NSSI); (d) The lethality of the gesture on a six points Likert scale where 0 = No physical damage or very minor physical damage; 1 = Minor physical damage (e.g., lethargic speech; first-degree burns; mild bleeding; sprains); 2 = Moderate physical damage; medical attention needed (e.g., conscious but sleepy, somewhat responsive); 3 = Moderately severe physical damage; medical hospitalization and likely intensive care required; 4 = Severe physical damage; medical hospitalization with intensive care required; 5 = Death. In the present study the variable “lethality of suicide attempt” is dichotomize in “low lethality” from no (0) to moderate (2) physical damage, and high lethality, from moderately severe (3) to severe (4) When more than one suicide attempt occurred, lethality is referred to the worst episode. The C-SSRS scores are assigned over a period ranging from three months (for suicidal ideation) and six months (suicide attempt) prior its administration. The the suicidal measures used as dependent variables of this study (suicide attempt, number of suicide attempts and potential lethality of the suicide attempt) cumulate observations that cover a period from three months prior the admission to six months after the admission. The C-SSRS psychometric properties, validity and satisfactory internal consistency (Cronbach’s alpha = 0.937) have been published. The scores were obtained after the administration of the’specific semi-structured clinical interview.

*Schedule for Affective Disorders and Schizophrenia for School Age Children, Present and Lifetime* (K-SADS-PL, [95]) is a semi-structured clinical interview used to assess current and past psychopathological features and psychiatric disorders in children and adolescents according to the Diagnostic and Statistical Manual of Mental Disorders, Fifth Edition (DSM-5), criteria. All patients and at least one of their parents or legal tutors were interviewed. This interview was used to identify the presence of MDD.

*Structured Clinical Interview for DSM-5 Personality Disorders* [96] is a semi-structured clinical interview that assesses the presence/absence of the 10 Personality Disorder according to DSM-IV-TR criteria. The Italian version of SCID-5-PD has good psychometric features: intraclass correlation coefficient (ICC) values ranged from 0.88 (Dependent PD and Histrionic PD) to 0.94 (Avoidant PD) for dimensional SCID-II interview dimensional ratings (median ICC value = 0.94). Cohen k values were also adequate for SCID-II interview categorical PD diagnoses (median k value = 0.89, SD = 0.11) [97]. The presence of a PD diagnosis was scored when the subject passed the diagnostic threshold for one of the 10 PDs; in the present study the variable “PD categorical overall” was scored when the subject received at least one categorical diagnosis for any of the 10 PDs. The dimensional scores for each PD were obtained by summing the number of criteria met by each subject for any of the 10 PDs. The variable PD dimensional overall was obtained by summing all the PDs criteria met by each subejct.

### Statistical analysis

Non-parametric bivariate correlations were used to test the significance of the associations between the suicidality variables (suicidal ideation, suicidal behavior, number and lethality of suicide attempts) and MDD, PD dimensional diagnosis, BPD dimensional diagnosis, demographic variables and other risk factors variables as assessed through the window of observation of the study.

To test the significance of MDD and PD categorical diagnosis (binary) or dimensional diagnosis as predictors of suicidal behavior and potential lethality, four separate logistic regressions with suicidal behavior or potential lethality as dependent variable, and MDD and PD categorical diagnosis (binary) or dimensional diagnoses as independent variables, and age and gender as covariates, were carried out. Logistic regression analyses were also employed to test the significance of BPD categorical diagnosis or dimensional as independent variables with suicidal behavior or potential lethality as dependent variable.

In order to evaluate the effect of MDD and PD categorical and dimensional diagnoses and BPD categorical and dimensional diagnoses on number of suicide attempts, four separate mixed-effects Poisson regression analyses were carried out to predict the frequency of suicide attempts and potential lethality of attempt as dependent variables and MDD, PD or BPD dimensional diagnoses as independent variables. Correlational and logistic regression analyses were performed using SPSS for Windows version 26, while STATA version 17 was used to carry out mixed-effects Poisson regressions.

All the predictors included in the logistic regressions and Poisson regression had showed significant positive correlations with the three suicidal variables investigated as outcome measures, with only two exceptions. Age and sex were included at step one in the regression models even if they did not show significant correlations with the suicidal variables, given the importance that age and sex assume in the literature on suicidality presented in the introduction. PD categorical was included in the regression model for suicidal potential lethality, since its correlation with potential lethality was not significant but showed a statistical tendency (*p* = 0.08) and also due to the statistical significant association it showed with the other two outcome suicidal variables.

## Results

Descriptive analysis of the independent variables examined in the study are shown in Table 1. None of the *ideators* at admission (*n* = 52) attempted suicide during the six month period of the follow-up of clinical observation, while only two patients among the *attempters* at admission (*n* = 66) made a subsequent suicide attempt (one and two suicide episodes, respectively, each one screened with the C-SSRS). Among the attempters, 51 (77,3%) made a low lethality suicidal attempt, and 15 (22,7%) made a high lethality suicidal attempt. The number of suicide attempts reported was one for 47 (71.2%) adolescents, two for 10 (15,2%) adolescents, three for 7 (10,6%) adolescents. Furthermore 2 (3%) adolescents reported each four and five suicide attempts.

**Table 1:** Demographic, diagnostic and risk profile features of the study sample (*N* = 118)

Variable	*n*	*%*
Female	90	76.3
Neglect	5	4.5
Any sexual abuse	8	7.1
Physical abuse	0	0
Mood Disorder	78	66.1
Anxiety Disorder	46	39.3
Eating Disorder	10	8.5
Substance Misuse	29	24.8
Borderline PD	21	17.8
Paranoid PD	1	0.8
Avoidant PD	6	5.1
Schizotypal PD	0	0
Narcissistic PD	2	1.7
Obsessive-Compulsive PD	0	0
Dependent PD	1	0.8
Histrionic PD	1	0.8
Schizoid PD	0	0
Attempters	66	55.9
	*Mean*	*SD*
Age	15.9	1.11
PD dimensional^a^	6.39	4.44

^a^Number of positive PD criteria met

### Correlational analysis

Bivariate correlational analysis revealed significant association, as shown in Table 2.

**Table 2:** Correlations between suicidality variables, demographic, psychopathological risk factors and diagnostic variables (*N* = 118)

Variables	Suicidal behavior	Suicidal ideation severity	Suicidal ideation intensity	Number suicide attempts	Suicide attempt lethality
Age	.11	.05	0.1	.07	.14
Gender	-.13	.02	0.1	-.05	-.13
Major Depressive Disorder	.25**	.24**	.31**	.27**	.19*
PD categorical	.18*	.06	.03	.25**	.15
PD dimensional	.19*	.01,	.04	.30**	.24**
Borderline PD	.22*	.05	.00	.25**	.19*
Borderline dimensional	.23**	.05	-.00	.29**	.22*
Narcissistic dimensional	.11	-.14	-.10	.12	.32**

^*^P < .05

^**^P < .001

### Predictor analyses

The first logistic regression model revealed that MDD was a significant predictor of suicidal behavior after controlling for age and gender (β_(1)_ = 1.32, *SE* = 0.44, *p* = 0.002) and it accounted for 15% of the total variance (Negalkerke *R*^2^ = 0.145). Adding diagnosis of PD binary to the equation made a significant contribution to the model (χ^2^_(1)_ = 4.11, *p* = 0.045) and increased the model fit to the data (Negalkerke *R*^2^ = 0.186). The diagnosis of PD made a further significant contribution to the prediction of suicidal behavior (β_(1)_ = 0.97, *SE* = 0.49, *p* = 0.049). Adding diagnosis of PD binary to the equation made a significant contribution to the model (χ^2^_(4)_ = 17.66, *p* = 0.001) and increased the model fit to the data (Negalkerke *R*^2^ = 0.186). Within the general model, the diagnosis of PD binary made a significant contribution to the increase in prediction of suicidal behavior (β_(1)_ = 0.97, *SE* = 0.49, *p* = 0.049). Although MDD remains the most significant predictor within the model with an ODD RATIO of 3.71, 95% CI 1.56, 8.87, the presence of PD binary diagnosis contributes to increase risk of suicidal behavior (ODD RATIO = 2.60, 95% CI 1.01, 6.89).

The second logistic regression showed that MDD was not a significant predictor of the lethality of suicide attempt (β_(1)_ = 0.69, *SE* = 0.51, *p* = 0.178; Negalkerke *R*^2^ = 0.111). Adding PD binary to the equation improved the significance of the model (χ^2^_(1)_ = 3.68, *p* = 0.055) and the percentage of the variance accounted for (Negalkerke *R*^2^ = 0.153). PD binary was a marginally significant predictor of lethality of suicide attempt (β_(1)_ = 0.97, *SE* = 0.50, *p* = 0.054). However, presence of PD binary increases the odds of greater lethality of suicidal behavior (OR = 2.63, 95% CI 0.98, 7.08) compared to MDD (OR = 2.09, 95% CI 0.78, 5.61).

The mixed-effects Poisson regression revealed that both MDD and PD binary were significant predictors of number of suicide attempts (β_(118)_ = 0.80, *SE* = 0.28, *z* = 2.92, *p* = 0.003, and β_(118)_ = 0.65, *SE* = 0.22, *z* = 2.95, *p* = 0.003, respectively). Presence of either MDD or PD binary diagnoses predicts double the number of suicide attempts compared to subjects with no MDD and PD binary diagnoses. When both MDD and PD binary are included in the model the number of suicide attempts is nearly predicted to be five times higher compared to no diagnoses of MDD and PD.

We repeated all the analyses with MDD and BPD binary diagnosis as predictor variables. We found that adding BPD binary diagnosis substantially improved the model for predicting suicidal behavior (χ^2^_(1)_ = 7.57, *p* = 0.006). BPD binary was found a significant predictor of suicidal behavior (β_(1)_ = 1.58, *SE* = 0.62, *p* = 0.011). The odds of occurrence of suicidal behavior associated with BPD diagnosis were 4.84 (95% CI 1.44, 16.23).

While MDD did not significantly impact on the significance of the predictive model for lethality of suicide attempt (χ^2^_(1)_ = 2.28, *p* = 0.131), BPD binary diagnosis significantly improved the model when added to the equation (χ^2^_(1)_ = 4.65, *p* = 0.030). BPD binary diagnosis was found to be a significant predictor of lethality (β_(1)_ = 1.21, *SE* = 0.56, *p* = 0.030) while MDD was not a significant predictor (β_(1)_ = 0.80, *SE* = 0.51, *p* = 0.119). Having a BPD binary diagnosis increased the odds of a lethal suicidal attempt to 3.36 (95% CI 1.16, 10.04).

The mixed-effects Poisson regression revealed that both MDD and BPD binary were highly significant predictors of number of suicide attempts (β_(118)_ = 0.88, *SE* = 0.27, *z* = 3.23, *p* = 0.001, and β_(118)_ = 0.78, *SE* = 0.24, *z* = 3.29, *p* = 0.001, respectively). The estimated marginal means show that the number of attempted suicides nearly trebles when either MDD and BPD binary are present, and it becomes six time higher when both diagnoses are present compared to subjects with no MDD and BPD.

Subsequently, we repeated the analyses with PD and BPD as dimensional diagnoses to test whether these increased the significance of the prediction. The stepwise logistic regression with suicidal behavior as dependent variable, MDD and PD dimensional score (defined as number of criteria met) as independent variables, showed that adding PD dimensional diagnosis at the final step substantially increased the significance of the model (χ^2^_(1)_ = 4.97, *p* = 0.026) and the model fit to the data (Negalkerke *R*^2^ = 0.194). PD dimensional diagnosis was found to be a significant predictor of suicidal behavior (β_(1)_ = 0.11, *SE* = 0.05, *p* = 0.031).

The second stepwise logistic regression with potential lethality as dependent variable, showed that adding PD dimensional score at the final step also further increased the significance of the model (χ^2^_(1)_ = 5.76, *p* = 0.016) and accounted for a greater share of the variance (Negalkerke R^2^ = 0.176). PD dimensional was a significant predictor of lethality of attempted suicide (β_(1)_ = 0.12, *SE* = 0.05, *p* = 0.020).

The mixed-effects Poisson regression revealed that PD dimensional diagnosis was a highly significant predictor of number of suicide attempts (β_(118)_ = 0.10, *SE* = 0.02, *z* = 4.26, *p* = 0.001).

Adding BPD dimensional score at the final step to the model construction with suicidal behavior as dependent variable substantially increases its significance (χ^2^_(1)_ = 9.94, *p* = 0.016) and the variance accounted for (Negalkerke *R*^2^ = 0.241). BPD dimensional was score revealed to be a significant predictor of suicidal behavior (β_(1)_ = 0.32, *SE* = 0.11, *p* = 0.003).

BPD dimensional score also improves the prediction when added to the model of potential lethality (χ^2^_(1)_ = 4.30, *p* = 0.038). BPD dimensional score was found to be a significant predictor of potential lethality of attempted suicide (β_(1)_ = 0.22, *SE* = 0.11, *p* = 0.042).

Finally, BPD dimensional was revealed a highly significant predictor of number of suicide attempts in the mixed-effects Poisson regression analysis (β_(116)_ = 0.18, *SE* = 0.05, *z* = 3.80, *p* = 0.001).

We also tested the impact of the number of narcissistic traits on the three suicide dependent variables. A significantly positive correlation emerged between NPD dimensional and the potential lethality of suicide attempt, but the NPD dimensional variable was not included in the regression models for potential lethality, given that only 2 subjects of the sample were diagnosed with NPD categorical diagnosis.

## Discussion

In this paper we evaluated the relative predictive strength of MDD diagnosis and PD categorical and dimensional diagnoses, independently and in combination, for suicidal risk in adolescence. Both the role of specific personality disorders BPD and NPD (the latter only as far as lethality of the suicide attempts is concerned), and personality disorder diagnosis overall, either as categorical or as dimensional constructs, were considered in the analyses.

Firstly, the results of this study confirm that MDD is a significant independent predictor of suicide attempts [27], but not for the suicide attempt potential lethality. Secondly, our results reveal that meeting criteria for any PD categorical diagnosis is a significant predictor of suicide attempt and of its potential lethality. Furthermore, the number of personality disorder criteria (dimensional diagnosis of PD) met is a significant predictor of the risk of suicide attempt and of its potential lethality. Besides showing a direct independent predictive effect on suicidal conducts, both categorical and dimensional diagnoses of PD significantly added to the variance explained by MDD, highlighting a cumulative impact of the affective disorder and personality pathology on suicidal behaviors in this sample.

To our knowledge, this is the first study to show the relevance of personality pathology overall for the prospective suicidal risk in adolescence [32]. Regarding the reliability of personality diagnoses in adolescence, current research indicates that PD diagnoses in adulthood show the same level of temporal instability as also found in adolescent diagnoses [81, 82, 98]. Even though categorical diagnoses of PDs in adulthood can be as unstable as the diagnoses obtained in adolescence, it was found that the more severe the personality pathology, the more stable the diagnosis is [99]. As said in the introduction, the continuity of personality pathology is attributed to core dimensions that are stable during development [81, 82]. The continuity of these core dimensions is due to both genetic and early environmental influences [81, 98]. These pathological personality traits can be accurately assessed in adolescence as outlined in the Alternative Model for Personality Disorders (AMPD) of the Section III of the DSM-5 [84, 85] and these traits hold for the homotypic continuity of personality diagnoses in the lifespan [70]. On the other hand, the observed instability of the PD diagnoses is currently interpreted as the product of the more transient nature of the psychopathological symptoms (internalizing and externalizing dimensions) included as criteria of PDs in the diagnostic manuals [82, 100, 101]. Thus, the relative instability of personality diagnoses in adolescence over time has been attributed to the variations of such behavioral manifestations due to the peculiarities of adolescence brain maturation as well as to the onset of externalizing and internalizing psychopathology [102]. When considering the aspect of prevention and early interventions in the developmental age, the importance of diagnosing personality disorders in adolescence cannot be underestimated. These diagnoses are reported to have a considerable impact on adolescent mental health services and professionals, given the association of PDs with several maladaptive behaviors and their impact on health in general [90]. Overall, it has been suggested that the extension of personality pathology can account and be considered a measure of a latent factor expressing a wider vulnerability to the development of psychopathology [84]. The results from this study seem to bolster this approach in confirming the importance of disturbance of personality at large as a general indicator of psychopathological vulnerability and maladaptive functioning in adolescence [29, 65].

When considering specific PD diagnoses, this study confirms that BPD is the most powerful predictor of attempted suicide risk and of the number of attempts at suicide. In particular, both categorical and dimensional approaches to diagnoses show BPD to be as significant independent predictor of suicidality as MDD. This is consistent with previous epidemiological observations where depression, personality pathology and suicidality were concurrently studied [56, 57]. In interpreting the independent and cumulative role of MDD and BPD, it should be considered that a significant debate has pointed to the possible overlapping between affective disorders and BPD. These diagnostic entities have been considered by some authors as clinical variants of the same temperamental or psychopathological spectrum [103, 104]. Some considerations can be made that contrast this view. Although literature report a high rate of comorbidity between affective disorders and BPD as well as a clear impact of BPD on recurrent depressive episodes, epidemiological data and the analysis of the clinical features of BD, MDD and BPD exclude the psychopathological overlapping between these clinical conditions [54, 105, 106]. As far as the issue of suicidality is concerned, empirical literature suggest that the distinct and cumulative predictive role of BPD and MDD should be attributed to the single clinical features pertaining each diagnostic entity that should be kept separate in order to enhance the predictability of the assessment of the sucidal risk [58–60].

Notably, in this study the categorical diagnosis of BPD and the number of borderline disorder symptoms exert a powerful cumulative effect on suicidality in the presence of MDD. This result seems to further support the need to evaluate the interaction between personality pathology, in particular BPD, and affective disorders in the suicidal process in adolescence as well as other maladaptive outcomes [74]. Importantly, we found no evidence of moderation effects in any of the models investigated: BPD diagnosis or BPD criteria met did not interact with MDD to amplify the impact of depression on suicidality. Rather, the risk factors are somewhat overlapping and additive, suggesting that the processes through which they increase the risk of suicidality in adolescents may be different: a possibility to be explored is that MDD creates vulnerability through a background of negative mood and hopelessness, while PD operates via impulsivity and affect dysregulation.

These results are consistent with the observations reported in the ‘National Epidemiologic Survey Mental disorders and risk of suicide attempt: on Alcohol and Related Conditions (NESARC)’ by Hoertel and colleagues [107]. These authors generated a bifactor model which parses disorder variance into general variance (i.e., variance of the general psychopathology factor), variance of dimensions of psychopathology (e.g. variance of the externalizing dimension) and unique variance (variance of each mental disorder per se) [108]. In this model the general psychopathology factor accounted for the prediction during a 3-year follow-up period of suicide attempts in a large general population sample (*n* = 35,000). Depression was an independent predictor only for females. Recent analysis of this and other datasets have shown that the general psychopathology factor and a latent BPD dimension correlate at *r* > 0.8 and could be considered a unitary construct rather than two separate entities [109]. Other independent investigations have also linked BPD criteria to a general psychopathology factor rather than an independent personality diagnosis [98, 110]. While obtained in a much smaller sample, the associations observed in our study may add to the notion that the risk of suicide is part of a general vulnerability to mental disorder [84, 111].

## Limitations

Although the study has areas of strength (it includes a clinical population, a longitudinal window of observation of suicidal behaviors, interview-based assessment,), a number of limitations need to be borne in mind when considering the results. Firstly, the sample is relatively small and not balanced for gender, although it is known that females have higher rates of attempted suicide while [11] successful suicides are more common in males than female [6]. Further, the sample may not be representative of the general population, as the adolescents participating were referred for a treatment setting offering relatively long-term day-hospital or inpatient interventions, so the findings may not be generalizable to milder presentations where such a treatment would not be indicated. Moreover, the lack of occurrence of suicide attempts in the 6 months period of follow-up could have been influenced by the *regime of treatment* being administered to all patients after their first admission. We are aware that this outcome may have had an impact on the perspective interpretation of the results.

As far as the interaction between MDD and personality pathology in heightening suicidal risk, we relied on a categorial assessment of MDD, but the actual strength of such interaction should also be proved introducing a dimensional measure of depressive symptoms as well.

Finally, while we incorporated more recent dimensional thinking into our approach to assessing psychopathology, our study still relied on traditional defined categories represented in Section II of the DSM. Future studies of prospective relations with suicide outcomes should include AMPD-defined personality pathology in adolescence to truly represent dimensional thinking.

Overall, the results of this study show that the effects of categorical and dimensional diagnoses of personality disorders on suicidal behaviors in adolescence are highly significant and have a strong additive effect when a diagnosis of MDD is present. As discussed in the introduction, our results give an empirical basis as to the importance of making a diagnosis of PD in adolescence, as it guides and improves the management of concurrent maladaptive behaviors, and, in particular, suicidality. It should also be noted that, in keeping with what already published in the clinical and empirical literature, the number of criteria the personality diagnoses considered in this study enhances the likelihood of suicidal risk in more significant way than the categorical diagnoses alone. This is a critical aspect of tis study pointing to the advantage of evaluating the severity of personality pathology for the management of suicidal risk in adolescence.

## Conclusions

This is the first study to highlight that the presence of PD overall is a highly significant risk factor for suicidal behavior in adolescence. In particular, BPD as a dimensional construct was found a significant predictor of suicidality six months after intake assessment, confirming the important role BPD and personality pathology at large plays in suicidal behavior both in adulthood and adolescence [57, 70, 98, 112]. This finding further strengthens the importance of assessing personality pathology as a general factor of psychopathological vulnerability in adolescence for the purpose of improving suicide risk management.

A second noteworthy result from this study is that MDD and PD have not only proved to represent independent significant risk factors for suicidality in adolescence, but they also show a significant interactive cumulative impact on suicidal risk. The significance of the number of personality traits as moderator between MDD and number of suicides attempts further highlights the interplay between mood disorders and personality pathology in increasing suicide risk in adolescence. This evidence further confirms the need for a rigorous clinical assessment of both conditions by mental health professionals involved in suicidal risk management in this phase of human development [113].

These results point to the importance of early identification of personality disorders and that the accurate assessment of the degree of severity of personality pathological functioning is important in shaping the clinical management and the treatment planning of services to lower suicidal risk in adolescence.

## Data Availability

The datasets generated and analyzed during the current study are not publicly available due the sensitivity of the matter under investigation, but are available from the corresponding author on reasonable request.
